# Predictors of all‐cause mortality among patients hospitalized with influenza, respiratory syncytial virus, or SARS‐CoV‐2

**DOI:** 10.1111/irv.13004

**Published:** 2022-05-24

**Authors:** Mackenzie A. Hamilton, Ying Liu, Andrew Calzavara, Maria E. Sundaram, Mohamed Djebli, Dariya Darvin, Stefan Baral, Rafal Kustra, Jeffrey C. Kwong, Sharmistha Mishra

**Affiliations:** ^1^ ICES Toronto Ontario Canada; ^2^ MAP Centre for Urban Health Solutions, Li Ka Shing Knowledge Institute, St. Michael's Hospital Unity Health Toronto Toronto Ontario Canada; ^3^ Centre for Clinical Epidemiology and Population Health Marshfield Clinic Research Institute Marshfield Wisconsin USA; ^4^ Department of Medicine University of Toronto Toronto Ontario Canada; ^5^ Department of Epidemiology John Hopkins Bloomberg School of Public Health Baltimore Maryland USA; ^6^ Dalla Lana School of Public Health University of Toronto Toronto Ontario Canada; ^7^ Public Health Ontario Toronto Ontario Canada; ^8^ Department of Family and Community Medicine University of Toronto Toronto Ontario Canada; ^9^ University Health Network Toronto Ontario Canada; ^10^ Centre for Vaccine Preventable Diseases University of Toronto Toronto Ontario Canada; ^11^ Institute of Health Policy, Management and Evaluation University of Toronto Toronto Ontario Canada; ^12^ Department of Medicine, St. Michael's Hospital Unity Health Toronto Toronto Ontario Canada; ^13^ Institute of Medical Sciences University of Toronto Toronto Ontario Canada

**Keywords:** hospitalization, influenza, mortality, respiratory syncytial virus, SARS‐CoV‐2

## Abstract

**Background:**

Shared and divergent predictors of clinical severity across respiratory viruses may support clinical and community responses in the context of a novel respiratory pathogen.

**Methods:**

We conducted a retrospective cohort study to identify predictors of 30‐day all‐cause mortality following hospitalization with influenza (*N* = 45,749; 2010‐09 to 2019‐05), respiratory syncytial virus (RSV; *N* = 24 345; 2010‐09 to 2019‐04), or severe acute respiratory syndrome coronavirus 2 (SARS‐CoV‐2; *N* = 8988; 2020‐03 to 2020‐12; pre‐vaccine) using population‐based health administrative data from Ontario, Canada. Multivariable modified Poisson regression was used to assess associations between potential predictors and mortality. We compared the direction, magnitude, and confidence intervals of risk ratios to identify shared and divergent predictors of mortality.

**Results:**

A total of 3186 (7.0%), 697 (2.9%), and 1880 (20.9%) patients died within 30 days of hospital admission with influenza, RSV, and SARS‐CoV‐2, respectively. Shared predictors of increased mortality included older age, male sex, residence in a long‐term care home, and chronic kidney disease. Positive associations between age and mortality were largest for patients with SARS‐CoV‐2. Few comorbidities were associated with mortality among patients with SARS‐CoV‐2 as compared with those with influenza or RSV.

**Conclusions:**

Our findings may help identify patients at greatest risk of illness secondary to a respiratory virus, anticipate hospital resource needs, and prioritize local prevention and therapeutic strategies to communities with higher prevalence of risk factors.

## 1 INTRODUCTION

The COVID‐19 pandemic has put tremendous strain on hospital systems, and exposed long‐standing issues in healthcare capacity.^1^ Knowing who is at highest risk of severe disease from respiratory viruses may support proactive clinical decision‐making and help distribute resources to healthcare settings with high prevalence of risk factors.^2^ This is particularly useful in the context of a new and emerging respiratory virus where information and resources are scarce.^2^, ^3^

Several studies have compared shared and divergent predictors of severe disease among patients with influenza and respiratory syncytial virus (RSV),^4^, ^5^, ^6^, ^7^, ^8^, ^9^ two respiratory viruses with high seasonal prevalence prior to the emergence of severe acute respiratory syndrome coronavirus 2 (SARS‐CoV‐2). However, few papers have compared predictors of severity across influenza, RSV, and SARS‐CoV‐2.

Communities are returning to pre‐pandemic contact and exposure patterns, which may increase the risk of all respiratory infections. At the same time, laboratory diagnostic testing is transitioning to pre‐pandemic approaches, where only a subset of hospitalized patients with viral respiratory or influenza‐like illness receive laboratory‐confirmed diagnoses.^10^ Thus, during periods of respiratory viral epidemics (particularly with novel emerging pathogens), shared predictors of severity across the clinically important respiratory viruses may: (1) reduce morbidity and mortality by prioritizing preventions (e.g., vaccinations), testing, and access to therapeutics (e.g., antivirals); and (2) prepare healthcare settings that will require greater resources based on the prevalence of the underlying predictors.

We conducted an observational study using extensive health administrative data from Ontario, Canada, to identify the direction and magnitude of shared and divergent predictors of 30‐day all‐cause mortality following hospitalization with influenza, RSV, or SARS‐CoV‐2 (prior to vaccine availability or variant emergence).

## 2 METHODS

### 2.1 Study setting and design

We conducted a retrospective cohort study of patients hospitalized with influenza, RSV, or SARS‐CoV‐2 using population‐based laboratory and health administrative data from Ontario, Canada (population 14.7 million).^11^ Ontario's healthcare system provides publicly funded physician services, laboratory testing, and hospital care for all residents with a provincial health card. Datasets used in this study were linked using unique encoded identifiers and analyzed at ICES.^12^

### 2.2 Case definitions and outcomes

#### 2.2.1 Hospitalizations

We generated three study cohorts to assess predictors of severe outcomes among patients hospitalized with influenza, RSV, and SARS‐CoV‐2, respectively. Patients with influenza and RSV were identified using hospitalization data from the Canadian Institute for Health Information's Discharge Abstract Database (DAD) during the 2010–2011 to 2018–2019 respiratory virus seasons. DAD captures administrative, clinical, and demographic information on all hospital discharges in Canada. Patients were considered hospitalized with influenza if their discharge abstract contained any of the following ICD‐10 codes: J09, J10.0, J10.1, J10.8, J11.0, J11.1, or J11.8. Patients were considered hospitalized with RSV if their discharge abstract contained any of the following ICD‐10 codes: J12.1, J20.5, J21.0, or B97.4. Case definitions were validated in Ontario against laboratory confirmation and showed high specificity (influenza: 98%; RSV: 99%) and positive predictive values (influenza: 91%; RSV: 91%).^13^

We used DAD, the Ontario Laboratories Information System (OLIS), and the Public Health Case and Contact Management System (CCM) to identify patients hospitalized with SARS‐CoV‐2 between March 1 and December 1, 2020. OLIS is an electronic repository of Ontario's laboratory test results, containing information on laboratory orders, patient demographics, provider information, and test results. CCM is a central data repository for all COVID‐19 case management, contact management, and reporting in Ontario. Patients were considered hospitalized with SARS‐CoV‐2 if: (1) they were documented as hospitalized in DAD and had a positive polymerase chain reaction test for SARS‐CoV‐2 within 14 days before or 3 days after hospital admission; or (2) they were documented as hospitalized in CCM.

#### 2.2.2 Mortality

Our primary outcome of interest was 30‐day all‐cause mortality following hospital admission with influenza, RSV, or SARS‐CoV‐2. We used the Registered Persons Database (RPDB) and CCM to identify patients who died within 30 days of hospital admission. RPDB contains basic demographic information including age, sex, postal code, and date of death among all residents with an Ontario health card.

### 2.3 Inclusion and exclusion criteria

**FIGURE 1:**
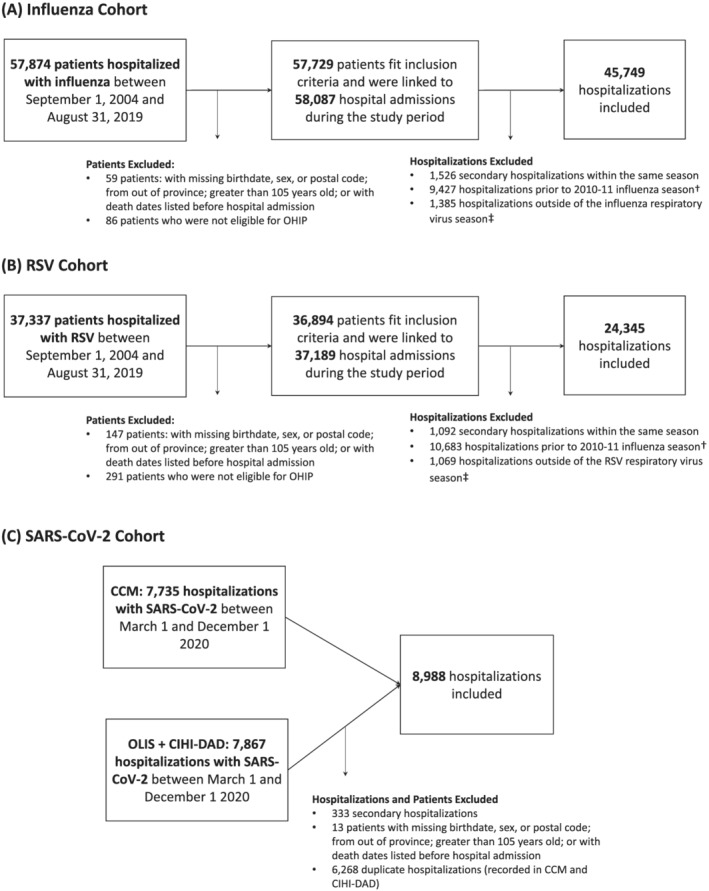
Study cohorts and exclusions. (A) Influenza hospitalization cohort. (B) RSV hospitalization cohort. (C) SARS‐CoV‐2 hospitalization cohort. Exclusions were made in the order in which they appear. RSV, respiratory syncytial virus; SARS‐CoV‐2, severe acute respiratory syndrome coronavirus 2; OHIP, Ontario health insurance plan. ^†^Influenza and RSV hospitalizations prior to the 2010–11 respiratory virus season were excluded to reduce selection bias due to changes in testing behavior following the H1N1 influenza epidemic in 2009–2010. ^‡^Virus seasonality was defined as November through May, and November through April for influenza and RSV, respectively

Hospitalized patients were excluded if: they were not eligible for the Ontario Health Insurance Plan; their birthdate, sex, or postal code was missing from RPDB; their residential postal code was outside of Ontario; they were older than 105 years according to their birthdate in RPDB; or their recorded death date predated hospital admission (Figure 1). Only one hospitalization per patient was included (per season for influenza and RSV, and overall for SARS‐CoV‐2). Among patients hospitalized with influenza or RSV, we included the first hospital admission of the season. Among patients hospitalized with SARS‐CoV‐2, we included any hospitalization that resulted in death within 30 days of admission, or the first admission if no other admission was associated with 30‐day mortality. Variation in inclusion criteria were due to suspected differences in hospital admission and discharge behavior across virus cohorts. For example, early in the pandemic, evidence suggested that patients hospitalized with SARS‐CoV‐2 had a relatively high likelihood of readmission within 60 days of discharge.^14^ Patients hospitalized with influenza or RSV were excluded if they were hospitalized outside of the respective respiratory virus season. Respiratory virus seasonality was defined as November to May for influenza, and November to April for RSV to align with case definitions from Hamilton et al.,^13^ and to create the most inclusive time frame to capture seasonal virus activity in Ontario.^15^

### 2.4 Predictors of 30‐day all‐cause mortality

We selected potential predictors of 30‐day all‐cause mortality a priori. Variables were considered if they had documented or suspected associations with respiratory virus acquisition or severity, or healthcare access in peer‐reviewed, published literature.

#### 2.4.1 Demographic characteristics

We used data from RPDB to describe pertinent individual‐level demographic characteristics including age, sex, and residence in rural neighborhoods.^16^ Rural neighborhoods were defined as those outside commuting zones of population centers (i.e., centers with more than 10,000 residents).

We used aggregated 2016 Canadian census data to describe neighborhood‐level social determinants of health associated with risk of respiratory virus acquisition,^17^, ^18^, ^19^ access to care, and discrimination within health care settings^20^, ^21^ including income,^22^ household size,^23^ and “ethnic concentration”^24^ (herein referred to as percent racialized). Neighborhood‐level variables were categorized into quintiles (i.e., 1 = 20% of neighborhoods with lowest values; 5 = 20% of neighborhoods with highest values). Patients were assigned a quintile according to their residential postal code. We describe derivation of the neighborhood‐level determinants of health in depth in the supporting information.

#### 2.4.2 Underlying health conditions

Pertinent underlying health conditions included: asthma, chronic obstructive pulmonary disease (COPD), hypertension, cardiac ischemic disease, congestive heart failure, stroke, dementia or frailty, chronic kidney disease, advanced liver disease, and immunosuppression (i.e., patients with a cancer diagnosis in the past 5 years, human immunodeficiency virus, solid organ or bone marrow transplant, or another immunodeficiency condition).^25^, ^26^ We used validated case definitions and health administrative data to classify each individual‐level health condition. Case definitions and validity are described in detail in Table S1.

#### 2.4.3 Other covariates

Other predictors of severe outcomes included residence in a long‐term care home (LTCH),^27^, ^28^ and seasonal immunization against influenza.^29^, ^30^, ^31^ We used the Chronic Care Reporting System, pharmacist billing claims in the Ontario Drug Benefits Database (ODB), and physician billing claims in the Ontario Health Insurance Plan (OHIP) database to determine whether individuals resided in a LTCH. We used ODB and OHIP to identify patients vaccinated against influenza between October 1 of the season of hospital admission and 14 days prior to admission. Relevant vaccination claim codes and drug identification numbers are outlined in Table S2.

### 2.5 Statistical analyses

Data processing and analyses were conducted using SAS version 9.4 (SAS Institute, Cary, NC). Frequencies and proportions were used to describe the distribution of risk factors among individuals hospitalized with influenza, RSV, or SARS‐CoV‐2. Modified Poisson regression (i.e., Poisson regression with a robust error variance) was used to assess the association between predictors and 30‐day all‐cause mortality. Modified Poisson regression was used over logistic regression to estimate risk ratios and avoid misinterpretation of odds ratios obtained from logistic regression.^32^ We calculated unadjusted and adjusted relative risk of dying within 30 days of hospital admission per predictor among each hospitalization cohort. Adjusted models included all other predictors. We qualitatively compared the direction, magnitude, and 95% confidence interval of associations among respective cohorts of hospitalized patients to identify shared and divergent predictors of 30‐day all‐cause mortality.

## 3 RESULTS

### 3.1 Characteristics of patients hospitalized with influenza, RSV, or SARS‐CoV‐2

We observed 45,749 influenza hospitalizations, 24,345 RSV hospitalizations, and 8988 SARS‐CoV‐2 hospitalizations after applying inclusion and exclusion criteria (Figure 1). Patients hospitalized with RSV were younger than patients hospitalized with influenza or SARS‐CoV‐2 (median age RSV patients = 1 year; median age influenza patients = 71 years; median age SARS‐CoV‐2 patients = 70 years). Only 47% of RSV patients presented with at least one comorbidity as compared with 84% of influenza patients and 82% of SARS‐CoV‐2 patients. Table 1 compares additional characteristics of hospitalized patients by virus and 30‐day all‐cause mortality.

**TABLE 1:** Descriptive characteristics of patients hospitalized with influenza, respiratory syncytial virus, or SARS‐CoV‐2

Characteristic	Influenza		RSV		SARS‐CoV‐2	
	*n* (%)	Deaths (%)	*n* (%)	Deaths (%)	*n* (%)	Deaths (%)
**Total**	45 749	3186	24 345	697	8988	1880
**Demographics**						
Age group						
0–4	4863 (10.6)	11 (0.3)	17 157 (70.5)	20 (2.9)	65 (0.7)	0 (0)
5–19	2363 (5.2)	12 (0.4)	557 (2.3)	7 (1.0)	67 (0.8)	0 (0)
20–49	4466 (9.8)	95 (3.0)	421 (1.7)	19 (2.7)	1367 (15.2)	36 (1.9)
50–64	6519 (14.2)	305 (9.6)	969 (4.0)	59 (8.5)	2150 (23.9)	186 (9.9)
65–74	7030 (15.4)	466 (14.6)	1298 (5.3)	88 (12.6)	1674 (18.6)	347 (18.5)
75–84	9981 (21.8)	827 (26.0)	1837 (7.5)	190 (27.3)	1826 (20.3)	535 (28.5)
≥85	10 527 (23.0)	1470 (46.1)	2106 (8.7)	314 (45.1)	1839 (20.5)	776 (41.3)
Male sex	21 831 (47.7)	1551 (48.7)	12 603 (51.8)	302 (43.3)	4768 (53.1)	1029 (54.7)
Living in rural area	3818 (8.3)	260 (8.2)	2243 (9.2)	41 (5.9)	253 (2.8)	53 (2.8)
Long‐term care resident	3048 (6.7)	814 (25.5)	721 (3.0)	168 (24.1)	1355 (15.1)	620 (33.0)
Immunized against seasonal influenza	14 718 (32.2)	1083 (34.0)	4106 (16.9)	287 (41.2)	3077 (34.2)	694 (36.9)
**Underlying health conditions**						
Asthma	12 838 (28.1)	762 (23.9)	6630 (27.2)	190 (27.3)	1777 (19.8)	345 (18.4)
COPD	11 924 (26.1)	1183 (37.1)	2593 (10.7)	273 (39.2)	1098 (12.2)	342 (18.2)
Cardiac ischemic disease	9301 (20.3)	1044 (32.8)	1717 (7.1)	225 (32.3)	1063 (11.8)	341 (18.1)
Congestive heart failure	12 240 (26.8)	1507 (47.3)	2769 (11.4)	349 (50.1)	1609 (17.9)	540 (28.7)
Hypertension	28 811 (63.0)	2644 (83.0)	5379 (22.1)	580 (83.2)	5912 (65.8)	1590 (84.6)
Diabetes	16 036 (35.1)	1373 (43.1)	2828 (11.6)	295 (42.3)	3795 (42.2)	996 (53.0)
Dementia/frailty	11 149 (24.4)	1557 (48.9)	2451 (10.1)	368 (52.8)	2604 (29.0)	940 (50.0)
Stroke	3999 (8.7)	445 (14.0)	738 (3.0)	102 (14.6)	679 (7.6)	224 (11.9)
Chronic kidney disease	9729 (21.3)	1091 (34.2)	2007 (8.2)	250 (35.9)	2120 (23.6)	711 (37.8)
Immunosuppression	7222 (15.8)	578 (18.1)	1936 (8.0)	174 (25.0)	602 (6.7)	146 (7.8)
Advanced liver disease	1305 (2.9)	115 (3.6)	246 (1.0)	26 (3.7)	252 (2.8)	47 (2.5)
**Neighborhood‐level social determinants of health**						
Income quintile						
Missing	149 (0.3)	11 (0.3)	147 (0.6)	1–5^a^	67 (0.8)	10 (0.5)
1 (lowest income)	12 199 (26.7)	773 (24.3)	5967 (24.5)	202 (29.0)	2617 (29.1)	541 (28.8)
2	10 187 (22.3)	798 (25.0)	5162 (21.2)	164 (23.5)	2081 (23.2)	483 (25.7)
3	8802 (19.2)	597 (18.7)	4754 (19.5)	113 (16.2)	1823 (20.3)	404 (21.5)
4	7564 (16.5)	482 (15.1)	4527 (18.6)	110 (15.8)	1293 (14.4)	234 (12.5)
5 (highest income)	6848 (15.0)	525 (16.5)	3788 (15.6)	103–107^a^	1107 (12.3)	208 (11.1)
Household size quintile						
Missing	416 (0.9)	30 (0.9)	380 (1.6)	10 (1.4)	139 (1.6)	34 (1.8)
1 (smallest household size)	11 947 (26.1)	911 (28.6)	4614 (19.0)	202 (29.0)	1729 (19.2)	410 (21.8)
2	8319 (18.2)	636 (20.0)	4507 (18.5)	129 (18.5)	1168 (13.0)	253 (13.5)
3	6173 (13.5)	445 (14.0)	3512 (14.4)	105 (15.1)	905 (10.1)	205 (10.9)
4	9605 (21.0)	665 (20.9)	5821 (23.9)	155 (22.2)	2156 (24.0)	457 (24.3)
5 (largest household size)	9289 (20.3)	499 (15.7)	5511 (22.6)	96 (13.8)	2891 (32.2)	521 (27.7)
Percent racialized quintile						
Missing	420 (0.9)	27 (0.8)	328 (1.3)	7 (1.0)	98 (1.1)	19 (1.0)
1 (least percent racialized)	7035 (15.4)	512 (16.1)	3573 (14.7)	85 (12.2)	579 (6.4)	115 (6.1)
2	7482 (16.4)	540 (16.9)	4124 (16.9)	99 (14.2)	843 (9.4)	212 (11.3)
3	8179 (17.9)	601 (18.9)	4510 (18.5)	135 (19.4)	1355 (15.1)	340 (18.1)
4	9578 (20.9)	743 (23.3)	5039 (20.7)	174 (25.0)	1914 (21.3)	380 (20.2)
5 (most percent racialized)	13 055 (28.5)	763 (23.9)	6771 (27.8)	197 (28.3)	4199 (46.7)	814 (43.3)

Abbreviations: COPD, chronic obstructive pulmonary disease; RSV, respiratory syncytial virus; SARS‐CoV‐2, severe acute respiratory syndrome coronavirus 2.

^a^ Cells with less than five patients have been suppressed to prevent individual identification.

### 3.2 Common predictors of 30‐day all‐cause mortality

Patients hospitalized with SARS‐CoV‐2 had the highest crude 30‐day all‐cause mortality rate (SARS‐CoV‐2 crude mortality rate = 20.9%; influenza crude mortality rate = 7.0%; RSV crude mortality rate = 2.9%).

**FIGURE 2:**
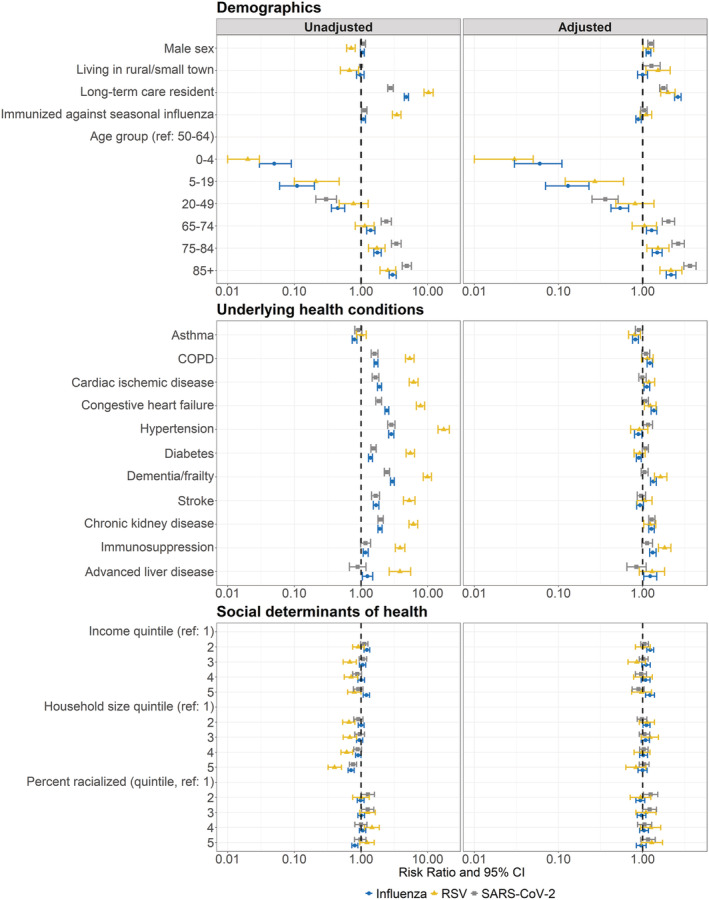
Unadjusted and adjusted predictors of 30‐day all‐cause mortality among patients hospitalized with influenza, RSV, or SARS‐CoV‐2. Modified Poisson regression was used to calculate associations between predictors and 30‐day all‐cause mortality. Adjusted models included all predictors. Influenza and RSV adjusted models additionally included season of hospital admission. Associations are presented as risk ratios (points) and 95% confidence intervals (error bars). RSV, respiratory syncytial virus; SARS‐CoV‐2, severe acute respiratory syndrome coronavirus 2; COPD, chronic obstructive pulmonary disease; CI, confidence interval

In unadjusted models, shared predictors of mortality included: older age, residence in a LTCH, immunization against seasonal influenza, COPD, cardiac ischemic disease, congestive heart failure, hypertension, diabetes, dementia/frailty, stroke, and chronic kidney disease (Figure 2, Table S3). Larger magnitudes of association between older age and mortality were observed among patients hospitalized with SARS‐CoV‐2 [unadjusted relative risk (RR) among 85+ vs. 50–64 = 4.88; 95% confidence interval (CI) = 4.16 to 5.72] versus influenza (unadjusted RR among 85+ vs. 50–64 = 2.99; 95% CI = 2.65 to 3.37) or RSV (unadjusted RR among 85+ vs. 50–64 = 2.53; 95% CI = 1.93 to 3.32). All other shared predictors of mortality showed larger magnitudes of association among patients hospitalized with RSV (Figure 2).

In adjusted models, shared predictors of mortality included: older age, male sex, residence in a LTCH, and chronic kidney disease (Figure 2, Table S4). Similar to unadjusted models, we observed larger magnitudes of association between older age and 30‐day all‐cause mortality among patients hospitalized with SARS‐CoV‐2 [SARS‐CoV‐2: adjusted RR (95% CI) 85+ vs. 50–64 = 3.64 (3.08 to 4.30); influenza: adjusted RR (95% CI) 85+ vs. 50–64 = 2.18 (1.91 to 2.48); RSV: adjusted RR (95% CI) 85+ vs. 50–64 = 2.16 (1.60 to 2.92)]. The magnitude and direction of associations between male sex, residence in a LTCH, and chronic kidney disease, and mortality were similar among patients hospitalized with all three viruses.

### 3.3 Notable differences among predictors of 30‐day all‐cause mortality

Rural residence was associated with increased 30‐day all‐cause mortality among patients hospitalized with RSV (adjusted RR = 1.52, 95% CI = 1.09 to 2.12) and SARS‐CoV‐2 (adjusted RR = 1.27, 95% CI = 1.01 to 1.61), but not among patients hospitalized with influenza (adjusted RR = 1.00, 95% CI = 0.87 to 1.14). Immunization against seasonal influenza was associated with decreased 30‐day all‐cause mortality among patients hospitalized with influenza (adjusted RR = 0.89, 95% CI = 0.83 to 0.96) but not patients hospitalized with RSV (adjusted RR = 1.09, 95% CI = 0.93 to 1.28) or SARS‐CoV‐2 (adjusted RR = 1.04, 95% CI = 0.95 to 1.13). Finally, cardiac ischemic disease, congestive heart failure, dementia/frailty, and immunosuppression were associated with increased all‐cause mortality among patients hospitalized with influenza and RSV after adjustment for all other predictors, but not among patients hospitalized with SARS‐CoV‐2.

## 4 DISCUSSION

We identified shared and divergent predictors of mortality among patients hospitalized with influenza, RSV, or SARS‐CoV‐2 using population‐based health administrative data from Ontario, Canada. In multivariable models, common predictors of 30‐day all‐cause mortality following hospitalization included older age, male sex, residence in a LTCH, and chronic kidney disease.

Older age and male sex were predictive of increased mortality across all respiratory virus cohorts, which aligns with numerous studies from high‐income countries^4^, ^5^, ^6^, ^7^, ^8^, ^9^, ^33^, ^34^, ^35^ and confirms the need to consider age and sex in clinical practice. The magnitude of association between older age and mortality was largest among patients with SARS‐CoV‐2, confirming robust evidence that age is an important predictor of severity among COVID‐19 patients, and should be used to guide targeted COVID‐19 preventions and therapeutics.^35^, ^36^

Residence in a LTCH was also a common predictor of 30‐day all‐cause mortality; however, associations were weaker among patients hospitalized with SARS‐CoV‐2. Differences in magnitudes of association may be due to greater selection bias of LTCH residents hospitalized with SARS‐CoV‐2 in comparison to those with influenza or RSV. For example, in Ontario, less than one quarter of COVID‐19‐positive LTCH residents were hospitalized prior to death, compared with nearly 80% of COVID‐19‐positive community residents during the first wave of the pandemic.^37^ It has been suggested that LTCH residents with SARS‐CoV‐2 may have been less likely to be hospitalized due to advanced care directives and/or informal policies that discouraged transfers of critically ill residents.^37^, ^38^, ^39^ The difference in hospitalizations prior to death narrowed in the pre‐vaccination second wave of the pandemic in Ontario,^37^ suggesting that the selection biases may have been specific to wave 1 and may not be reflective of past influenza or RSV seasonal epidemics.

Similar to previous studies,^40^ chronic kidney disease increased risk of 30‐day all‐cause mortality with similar magnitudes of effect among patients hospitalized with influenza, RSV, or SARS‐CoV‐2. Several other comorbidities were important predictors of mortality among patients with influenza or RSV, but not SARS‐CoV‐2 despite their known associations with SARS‐CoV‐2 severity.^26^ These comorbidities may have been associated with mortality among influenza and RSV patients, but not SARS‐CoV‐2 patients due to: (1) smaller sample size of the latter; (2) greater hospitalization rates of less severe patients with comorbidities who were infected with SARS‐CoV‐2 (i.e., selective hospitalization of patients with comorbidities due to limited understanding of the virus and disease trajectory); and/or (3) true clinical differences between patients requiring hospitalization with SARS‐CoV‐2 versus seasonal influenza or RSV.^41^, ^42^, ^43^ Moreover, age may act as an effect measure modifier on the relationship between comorbidities and mortality due to SARS‐CoV‐2. More research is needed to compare the immunological and clinical disease progression of influenza, RSV, and SARS‐CoV‐2 to better explain observed differences in risk by comorbidity.

We did not observe associations between area‐level social determinants of health and 30‐day all‐cause mortality following hospitalization with all three viruses, despite their associations with infection transmission risk.^17^, ^18^, ^19^, ^20^, ^21^ Lack of associations may be due to misclassification of neighborhood‐level social determinants of health (as these metrics were derived from the 2016 census), ecological fallacy, or adjustment of mediators in the causal pathway between income, household size, or racialization, and 30‐day all‐cause mortality.

This study is limited by potential misclassification of influenza and RSV cases, as we identified patients using their hospitalization discharge codes rather than diagnostic test results. However, case definitions were validated against a population of hospitalized patients who received diagnostic testing for influenza or RSV in the Ontario population.^13^ The case definitions had high specificity (influenza = 98%; RSV = 99%) and positive predictive values (influenza = 91%; RSV = 91%). Thus, misclassification of influenza and RSV hospitalization is likely rare. Moreover, the use of influenza and RSV case definitions allowed us to obtain hospitalization data across more respiratory virus seasons, increasing the generalizability of our findings.

Moreover, the study outcome as defined may capture deaths attributable to the virus or deaths in the context of an incidental infection (i.e., death *with* the virus). The additional public health dataset available for SARS‐CoV‐2 (i.e., CCM) allowed us to compare death outcomes of hospitalized patients with SARS‐CoV‐2 (this dataset documents whether cause of death was due to, or likely due to COVID‐19) to their mortality within 30 days of hospitalization in health administrative data. Thirty‐day all‐cause mortality had 97% positive predictive value against death due to COVID‐19 in CCM. Thus, we expect 3% of outcomes to potentially reflect deaths with SARS‐CoV‐2. In the absence of approaches to adequately address this misclassification for all viruses, we acknowledge the small bias as an important limitation.

This study is also limited by a lack of data on other important predictors of respiratory infection severity such as pregnancy,^44^, ^45^ obesity,^46^ and individual‐level social determinants (e.g., economic marginalization and racialization), which are known to mediate quality of hospitalized care and rates of respiratory virus infection.^17^, ^18^, ^19^, ^20^, ^21^ When using our results to inform prioritization of services, or to develop clinical prediction tools, we must consider these limitations so that other at‐risk patients do not fall through the cracks.

Finally, to provide insights on shared predictors of mortality in the context of a novel, emerging pathogen, we purposefully restricted the study period of SARS‐CoV‐2 to exclude hospitalizations of patients vaccinated against SARS‐CoV‐2, or those with SARS‐CoV‐2 variants. Future work would benefit from comparisons of predictors of mortality among patients hospitalized with influenza, RSV, or SARS‐CoV‐2 variants and/or breakthrough infections in the context of a vaccine to better inform responses to re‐emerging respiratory pathogens.

Our results add to the growing literature base comparing similarities and differences in clinical disease progression of patients hospitalized with influenza and SARS‐CoV‐2^41^, ^42^, ^43^ and have three important implications for clinical care and health systems. First, shared predictors of mortality could be used to identify, target, and prioritize hospitalized patients who are at greatest risk of death for prevention (e.g., vaccines), testing (e.g., rapid tests), and therapeutics (e.g., antivirals) in the context of a novel respiratory pathogen. Second, the underlying prevalence of shared predictors in a given geography could help prepare health systems for, and efficiently allocate health resources during, emergence of a novel respiratory pathogen. Finally, differences in observed predictors of mortality across the three viruses signal the importance of sufficient virus‐specific laboratory testing to ensure at‐risk individuals are not left behind.

## 5 CONCLUSION

We identified common predictors of 30‐day all‐cause mortality following hospitalization with SARS‐CoV‐2, influenza, or RSV in a population‐based cohort from Ontario, Canada. Shared predictors of mortality may help identify patients at greatest risk for syndromic clinical management of illness from respiratory viruses, anticipate local resource needs (e.g., for communities and hospitals), and prioritize prevention and therapeutic strategies during rapid emergence of respiratory viruses.

## CONFLICT OF INTERESTS

The authors declare no competing interests that are relevant to the content of this article.

## AUTHOR CONTRIBUTIONS

**Mackenzie A. Hamilton:** Conceptualization; formal analysis; investigation; methodology; visualization; original draft preparation. **Ying Liu:** Data curation. **Andrew Calzavara:** Data curation; methodology. **Maria E. Sundaram:** Conceptualization; formal analysis; investigation; methodology. **Mohamed Djebli:** Conceptualization; formal analysis; investigation; methodology. **Dariya Darvin:** Visualization. **Stefan Baral:** Supervision. **Rafal Kustra:** Formal analysis; methodology; supervision. **Jeff C. Kwong:** Conceptualization; funding acquisition; methodology; project administration; supervision. **Sharmistha Mishra:** Conceptualization; funding acquisition; methodology; project administration; supervision.

### PEER REVIEW

The peer review history for this article is available at https://publons.com/publon/10.1111/irv.13004.

## Supporting information


**Suppplementary Text**. Derivation of neighbourhood‐level social determinants of health.
**Table S1.** Definitions of underlying medical conditions among hospitalized patients.
**Table S2.** Databases and claim codes to identify patients immunized against seasonal influenza.
**Table S3.** Unadjusted predictors of 30‐day all‐cause mortality among patients hospitalized with influenza, respiratory syncytial virus, or SARS‐CoV‐2 (2020‐03 to 2020‐12).
**Table S4.** Adjusted predictors of 30‐day all‐cause mortality among patients hospitalized with influenza, respiratory syncytial virus, or SARS‐CoV‐2 (2020‐03 to 2020‐12).

## Data Availability

The dataset from this study is held securely in coded form at ICES. Although legal data sharing agreements between ICES and data providers (e.g., healthcare organizations and government) prohibit ICES from making the dataset publicly available, access may be granted to those who meet pre‐specified criteria for confidential access, available at www.ices.on.ca/DAS (email: das@ices.on.ca). The full dataset creation plan and underlying analytic code are available from the authors upon request, understanding that the computer programs may rely upon coding templates or macros that are unique to ICES and are therefore either inaccessible or may require modification.

## References

[1] Sen‐Crowe B , Sutherland M , McKenney M , Elkbuli A . A closer look into global hospital beds capacity and resource shortages during the COVID‐19 pandemic. J Surg Res. 2021;260:56‐63. doi:10.1016/j.jss.2020.11.062 33321393 PMC7685049

[2] Wynants L , Van Calster, B , Collins GS , et al. Prediction models for diagnosis and prognosis of COVID‐19: systematic review and critical appraisal. BMJ. 2020;369:26. doi:10.1136/bmj.m1328 PMC7222643 32265220

[3] Williams RD , Markus AF , Yang C , et al. Seek COVER: using a disease proxy to rapidly develop and validate a personalized risk calculator for COVID‐19 outcomes in an international network. BMC Med Res Methodol. 2022;22(1):1‐13. doi:10.1186/s12874-022-01505-z 35094685 PMC8801189

[4] Ackerson B , Tseng HF , Sy LS , et al. Severe morbidity and mortality associated with respiratory syncytial virus versus influenza infection in hospitalized older adults. Clin Infect Dis. 2019;69(2):197‐203. doi:10.1093/cid/ciy991 30452608 PMC6603263

[5] Atamna A , Babich T , Froimovici D , et al. Morbidity and mortality of respiratory syncytial virus infection in hospitalized adults: comparison with seasonal influenza. Int J Infect Dis. 2021;103:489‐493. doi:10.1016/j.ijid.2020.11.185 33249288

[6] Jansen AGSC , Sanders EAM , Hoes AW , van Loon AM , Hak E . Influenza‐ and respiratory syncytial virus‐associated mortality and hospitalisations. Eur Respir J. 2007;30(6):1158‐1166. doi:10.1183/09031936.00034407 17715167

[7] Kwon YS , Park SH , Kim MA , et al. Risk of mortality associated with respiratory syncytial virus and influenza infection in adults. BMC Infect Dis. 2017;17(1):785. doi:10.1186/s12879-017-2897-4 29262784 PMC5738863

[8] Zhang Y , Wang Y , Zhao J , et al. Severity and mortality of respiratory syncytial virus vs influenza A infection in hospitalized adults in China. Influenza Other Respi Viruses. 2020;14(5):483‐490. doi:10.1111/irv.12754 PMC7431648 32449300

[9] Cohen R , Babushkin F , Geller K , Finn T . Characteristics of hospitalized adult patients with laboratory documented influenza A, B and respiratory syncytial virus—a single center retrospective observational study. PLoS One. 2019;14(3):e0214517. doi:10.1371/journal.pone.0214517 30921408 PMC6438521

[10] Gostin LO . Life after the COVID‐19 pandemic. JAMA Heal Forum. 2022;3(2):e220323. doi:10.1001/JAMAHEALTHFORUM.2022.0323 36218829

[11] Statistics Canada . Table 17‐10‐0009‐01 Population Estimates, Quarterly.

[12] ICES Data and Privacy. Accessed January 12, 2022. https://www.ices.on.ca/Data-and-Privacy

[13] Hamilton MA , Calzavara A , Emerson SD , et al. Validating International Classification of Disease 10th Revision algorithms for identifying influenza and respiratory syncytial virus hospitalizations. PLoS One. 2021;16(1):1‐12. doi:10.1371/journal.pone.0244746 PMC7790248 33411792

[14] Donnelly JP , Wang XQ , Iwashyna TJ , Prescott HC . Readmission and death after initial hospital discharge among patients with COVID‐19 in a large multihospital system. Jama. 2021;325(3):304‐306. doi:10.1001/jama.2020.21465 33315057 PMC7737131

[15] Public Health Ontario . Ontario Respiratory Pathogen Bulletin. Accessed January 12, 2022. https://www.publichealthontario.ca/en/data-and-analysis/infectious-disease/respiratory-pathogens-weekly

[16] Wilson R , Rourke J , Oandasan IF , Bosco C . Progress made on access to rural health care in Canada. Can Fam Physician. 2020;25:66. doi:10.4103/CJRM.CJRM_84_19 31854338

[17] Upshaw TL , Brown C , Smith R , Perri M , Ziegler C , Pinto AD . Social determinants of COVID‐19 incidence and outcomes: a rapid review. PLoS One. 2021;16(3):e0248336. doi:10.1371/journal.pone.0248336 33788848 PMC8011781

[18] Foley D , Best E , Reid N , Berry M . Respiratory health inequality starts early: The impact of social determinants on the aetiology and severity of bronchiolitis in infancy. J Paediatr Child Health. 2019;55(5):528‐532. doi:10.1111/jpc.14234 30264506

[19] Martin LJ , Chen Y , Serrano‐Lomelin J , Talbot J , Yasui Y . Higher levels of social and material deprivation are associated with higher rates of influenza‐like illness‐related emergency department visits: Edmonton, Alberta. Public Health. 2020;189:117‐122. doi:10.1016/j.puhe.2020.06.039 33221645

[20] Egede LE , Walker RJ . Structural racism, social risk factors, and COVID‐19—a dangerous convergence for Black Americans. N Engl J Med. 2020;383(12):e77. doi:10.1056/NEJMp2023616 32706952 PMC7747672

[21] Siegel M , Critchfield‐Jain I , Boykin M , Owens A . Actual racial/ethnic disparities in COVID‐19 mortality for the non‐Hispanic black compared to non‐Hispanic White population in 35 US states and their association with structural racism. J Racial Ethn Heal Disparities. 2021;1‐13. doi:10.1007/s40615-021-01109-1 PMC8077854 33905110

[22] Statistics Canada . Total income. Dictionary, Census of Population, 2016. 2017. Accessed January 28, 2022. https://www12.statcan.gc.ca/census-recensement/2016/ref/dict/pop123-eng.cfm

[23] Statistics Canada . Private dwelling. Dictionary, Census of Population, 2016. 2017. Accessed January 28, 2022. https://www12.statcan.gc.ca/census-recensement/2016/ref/dict/dwelling-logements005-eng.cfm

[24] Matheson FI , van Ingen T. 2016 Ontario Marginalization Index: User Guide 2018. 10.17269/s41997-021-00552-1 PMC8975983 34432255

[25] Mertz D , Kim TH , Johnstone J , et al. Populations at risk for severe or complicated influenza illness: systematic review and meta‐analysis. BMJ. 2013;347(7923). doi:10.1136/bmj.f5061 PMC3805492 23974637

[26] Chidambaram V , Tun NL , Haque WZ , et al. Factors associated with disease severity and mortality among patients with COVID‐19: a systematic review and meta‐analysis. PLoS One. 2020;15(11):e0241541. doi:10.1371/journal.pone.0241541 33206661 PMC7673562

[27] Childs A , Zullo AR , Joyce NR , et al. The burden of respiratory infections among older adults in long‐term care: a systematic review. BMC Geriatr. 2019;19(1):1‐10. doi:10.1186/s12877-019-1236-6 31382895 PMC6683564

[28] Falsey AR , Treanor JJ , Betts RF , Walsh EE . Viral respiratory infections in the institutionalized elderly: clinical and epidemiologic findings. J am Geriatr Soc. 1992;40(2):115‐119. doi:10.1111/j.1532-5415.1992.tb01929.x 1740594 PMC7166501

[29] Chung H , Buchan SA , Campigotto A , et al. Influenza vaccine effectiveness against all‐cause mortality following laboratory‐confirmed influenza in older adults, 2010–2011 to 2015–2016 seasons in Ontario, Canada. Clin Infect Dis. 2021;73(5):e1191‐e1199. doi:10.1093/cid/ciaa1862 33354709 PMC8423473

[30] Bechini A , Ninci A , Del Riccio M , et al. Impact of influenza vaccination on all‐cause mortality and hospitalization for pneumonia in adults and the elderly with diabetes: a meta‐analysis of observational studies. Vaccine. 2020;8(2). doi:10.3390/vaccines8020263 PMC7349976 32486233

[31] Wilcox CR , Islam N , Dambha‐Miller H . Association between influenza vaccination and hospitalisation or all‐cause mortality in people with COVID‐19: a retrospective cohort study. BMJ Open Respir Res. 2021;8(1):e000857. doi:10.1136/bmjresp-2020-000857 PMC7934200 33664123

[32] Zou G . A modified Poisson regression approach to prospective studies with binary data. Am J Epidemiol. 2004;159(7):702‐706. doi:10.1093/aje/kwh090 15033648

[33] Stein RT , Bont LJ , Zar H , et al. Respiratory syncytial virus hospitalization and mortality: systematic review and meta‐analysis. Pediatr Pulmonol. 2017;52(4):556‐569. doi:10.1002/ppul.23570 27740723 PMC5396299

[34] Bonanad C , García‐Blas S , Tarazona‐Santabalbina F , et al. The effect of age on mortality in patients with COVID‐19: a meta‐analysis with 611,583 subjects. J am Med Dir Assoc. 2020;21(7):915‐918. doi:10.1016/j.jamda.2020.05.045 32674819 PMC7247470

[35] Biswas M , Rahaman S , Biswas TK , Haque Z , Ibrahim B . Association of sex, age, and comorbidities with mortality in COVID‐19 patients: a systematic review and meta‐analysis. Intervirology. 2021;64(1):36‐47. doi:10.1159/000512592 PMC7801974 33296901

[36] O'Driscoll M , Ribeiro Dos Santos G , Wang L , et al. Age‐specific mortality and immunity patterns of SARS‐CoV‐2. Nat. 2020;590(7844):140‐145. doi:10.1038/s41586-020-2918-0 33137809

[37] Brown KA , Daneman N , Buchan SA , Chan AK , Stall NM . Variation in care of community and nursing home residents who died of COVID‐19 in Ontario, Canada. J am Med Dir Assoc. 2021;22(6):1149‐1150. doi:10.1016/j.jamda.2021.04.008 33989538 PMC8057761

[38] Reith T. “No benefit” to sending seniors ill with COVID‐19 to hospital, some nursing homes tell loved ones. CBC News 2020. Accessed May 3, 2022. https://www.cbc.ca/news/health/covid-19-long-term-care-1.5519657

[39] Ontario Long Term Care Commission Meeting with Chartwell Retirement Residences . 2020. Accessed May 3, 2022. http://www.ltccommission‐commissionsld.ca/transcripts/pdf/Chartwell_Retirement_Residences_Transcript_October_09_2020.pdf

[40] Su G , Iwagami M , Qin X , et al. Kidney disease and mortality in patients with respiratory tract infections: a systematic review and meta‐analysis. Clin Kidney J. 2021;14(2):602‐611. doi:10.1093/ckj/sfz188 33623685 PMC7886553

[41] Piroth L , Cottenet J , Mariet AS , et al. Comparison of the characteristics, morbidity, and mortality of COVID‐19 and seasonal influenza: a nationwide, population‐based retrospective cohort study. Lancet Respir Med. 2021;9(3):251‐259. doi:10.1016/S2213-2600(20)30527-0 33341155 PMC7832247

[42] Laris‐González A , Avilés‐Robles M , Domínguez‐Barrera C , et al. Influenza vs. COVID‐19: comparison of clinical characteristics and outcomes in pediatric patients in Mexico City. Front Pediatr. 2021;9. doi:10.3389/fped.2021.676611 PMC8264261 34249813

[43] Hedberg P , Karlsson Valik J , Van Der Werff S , et al. Clinical phenotypes and outcomes of SARS‐CoV‐2, influenza, RSV and seven other respiratory viruses: a retrospective study using complete hospital data. Thorax. 2022;77(2):1‐10. doi:10.1136/thoraxjnl-2021-216949 PMC8260304 34226206

[44] Mertz D , Lo CKF , Lytvyn L , for the FLURISK‐Investigators , Ortiz JR , Loeb M . Pregnancy as a risk factor for severe influenza infection: an individual participant data meta‐analysis. BMC Infect Dis. 2019;19(1):1‐10. doi:10.1186/s12879-019-4318-3 31375073 PMC6679491

[45] Villar J , Ariff S , Gunier RB , et al. Maternal and neonatal morbidity and mortality among pregnant women with and without COVID‐19 infection: the INTERCOVID multinational cohort study. JAMA Pediatr. 2021;175(8):817‐826. doi:10.1001/jamapediatrics.2021.1050 33885740 PMC8063132

[46] Popkin BM , Du S , Green WD , et al. Individuals with obesity and COVID‐19: a global perspective on the epidemiology and biological relationships. Obes Rev. 2020;21(11):e13128. doi:10.1111/obr.13128 32845580 PMC7461480

